# A tissue-mimetic nano-fibrillar hybrid injectable hydrogel for potential soft tissue engineering applications

**DOI:** 10.1038/s41598-017-18523-3

**Published:** 2018-01-18

**Authors:** Neda Latifi, Meisam Asgari, Hojatollah Vali, Luc Mongeau

**Affiliations:** 1 Department of Mechanical Engineering, McGill University, 817 Sherbrooke street west, Montreal, QC H3A 0C3 Canada; 2 Department of Anatomy & Cell Biology, McGill University, 3640 University street, Montreal, QC H3A 2B2 Canada

## Abstract

While collagen type I (Col-I) is commonly used as a structural component of biomaterials, collagen type III (Col-III), another fibril forming collagen ubiquitous in many soft tissues, has not previously been used. In the present study, the novel concept of an injectable hydrogel with semi-interpenetrating polymeric networks of heterotypic collagen fibrils, with tissue-specific Col-III to Col-I ratios, in a glycol-chitosan matrix was investigated. Col-III was introduced as a component of the novel hydrogel, inspired by its co-presence with Col-I in many soft tissues, its influence on the Col-I fibrillogenesis in terms of diameter and mechanics, and its established role in regulating scar formation. The hydrogel has a nano-fibrillar porous structure, and is mechanically stable under continuous dynamic stimulation. It was found to provide a longer half-life of about 35 days than similar hyaluronic acid-based hydrogels, and to support cell implantation in terms of viability, metabolic activity, adhesion and migration. The specific case of pure Col-III fibrils in a glycol-chitosan matrix was investigated. The proposed hydrogels meet many essential requirements for soft tissue engineering applications, particularly for mechanically challenged tissues such as vocal folds and heart valves.

## Introduction

Considerable efforts have been made over the past few decades to develop scaffolding materials which mimic the extracellular matrix (ECM) for *Soft Tissue Engineering* (STE), the process of synthesizing natural tissue for the repair or replacement of diseased or lost tissues^1–6^. These scaffolding materials are used *in vivo*, for *in-situ* tissue regeneration, or *in vitro* for the fabrication of tissue substitutes in tissue culture bioreactors^7,8^, or as controlled tissue-mimetic microenvironments to investigate the effects of biomechanical and biochemical stimuli on cell behavior^2^. The chemical composition and microstructure of the scaffolds considerably influence tissue regeneration and function restoration. Scaffolds should be biocompatible and biodegradable with favorable structural, biochemical and biological properties^9^.

Injectable hydrogels, a class of highly hydrated polymer scaffolds, meet many of the criteria required for STE^10^, such as biocompatibility, biodegradability, low toxicity, high tissue-like water content and cell distribution homogeneity. Most injectable hydrogels are porous, which enhances the transfer of required nutrients and gases. The biomechanical properties of injectable hydrogels can be tuned for specific applications^4,11^. It is frequently hypothesized that cells encapsulated in the hydrogels sense their biomechanical microenvironment through focal adhesion. This is important for engineering mechanically active tissues such as vocal folds, heart valves and blood vessels, for which the scaffold provides the cells with effective biomechanical stimulation to produce and remodel neo-ECM^12,13^. Natural hydrogels have been extensively used for STE applications due to their resemblance in components and properties to natural ECM proteins. They yield excellent biocompatibility and bioactivity in comparison with synthetic materials^11^. Typical naturally derived hydrogels usually include two or more biopolymer-based materials, such as proteins (e.g., collagen (Col), gelatin (Ge), elastin and fibrin) and polysaccharides (e.g., chitosan, hyaluronic acid (HA) and alginate) in their intact or modified state^11^.

**Figure 1:**
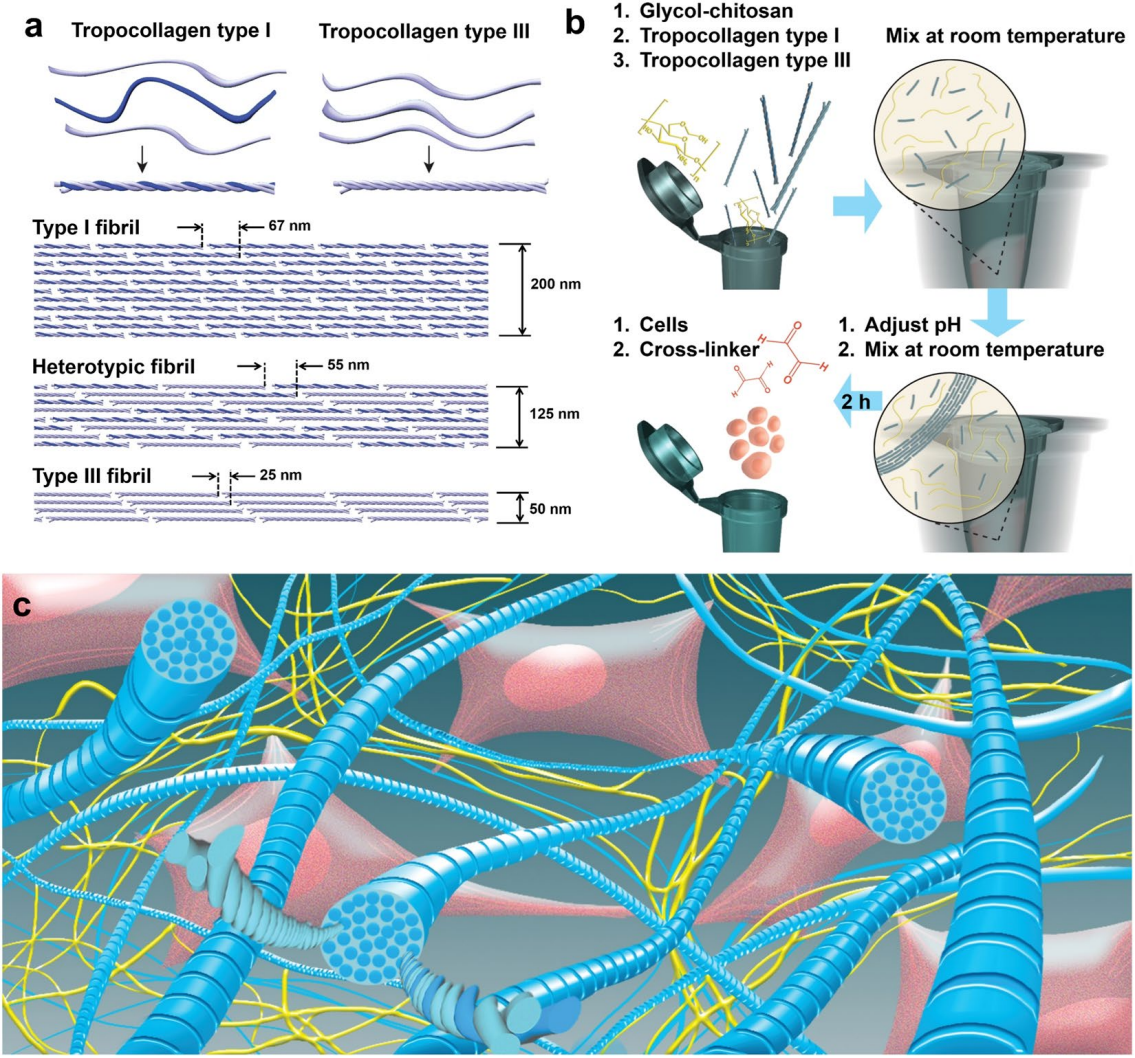
(**a**) Schematic of tropocollagen types I and III followed by their arrangements to form type I fibrils, heterotypic fibrils of types I and III (I&III), and type III fibrils. These illustrations are further supported by data reported in a recent study, in which average (fibril diameter, periodicity) of (200,67), (125,55) and (50,25) were obtained for types I, I&III with a mixing ratio of 1:1, and III fibrils, respectively^23^; (**b**) Schematic of the step-by-step fabrication procedure. Tropocollagen types I and III molecules were added to glycol-chitosan (GCS) solution, and the mixture was vortexed at room temperature. After adjusting pH to the physiological pH level, the mixture was vortexed again. At this stage, the mixture includes both tropocollagen molecules and newly-formed collagen fibrils. After 2 hours, cells were added and properly mixed. Finally, the cross-linker (glyoxal) was added, and the mixture was mixed to ensure a homogenous cell distribution; (**c**) Schematic of the three-dimensional structure of the nano-fibrillar hybrid hydrogel (Col-I&III/GCS). Heterotypic collagen fibrils (shown in blue) were randomly distributed in GCS matrix (shown in yellow). Heads of the tropocollagen molecules are shown on the cross-sections of the representative fibrils. Glyoxal was used to form covalent cross-linking between GCS molecules as well as between collagen fibrils and GCS matrix. The proposed hydrogel supports cell adhesion because of cell attachments to collagen fibrils, as illustrated (Col-I&III: the simultaneous presence of Col-I and Col-III).

Collagen is involved in the development and regeneration of various soft tissues^14–18^. It also plays a crucial role in tissues’ mechanical and biological properties. Fibril-forming collagens such as types I and III (Fig. 1a) contribute to the structural framework of various human tissues^14,16,19^. Collagen type I (Col-I), the most widely found collagen in the human body, forms thick collagen fibrils and fiber bundles in many soft tissues such as those of the heart, tendons, skin, lungs, cornea, vocal folds and vasculature^14,16,20–23^. This collagen type is the major support element of connective tissues, showing minimal distensibility under mechanical loading^24^. Collagen-based scaffolds, incorporating collagen types I or II as the key constituent, have been frequently investigated for *in vivo* applications such as wound dressing, dermal filling and drug/gene delivery^22,25–27^ as well as a wide range of *in vitro* applications^28–30^, due to collagen’s excellent biocompatibility, biodegradability, low immunogenicity, biological properties, and its role in tissue formation^7,18,22,31,32^. The long-term exposure to collagen-based biomaterials containing Col-I might yield progressive scarring based on the published literature^33^.

Collagen type III (Col-III) is widely present in tissues that contain Col-I, i.e., embryonic skin, lungs and blood vessels, as well as in distensible tissues such as vocal folds, the bladder and the uterus^23,34–37^. Although the biochemical composition of Col-III is not significantly different from that of Col-I, a certain amount of Col-III was previously demonstrated to be necessary to maintain tension and contraction development in several tissues^38–40^. For instance, a low ratio of Col-III to Col-I in bladder tissue was shown to alter fibril size distribution, and thus to yield greater compliance and less effective neurotransmitter function^39^. Previous studies have also shown that Col-III is formed developmentally and during wound healing^34,39–44^. Col-III has been previously demonstrated to modulate scar formation^45–48^. Humans with Col-III deficiency have impaired wound healing, often accompanied by excessive scar formation^23,48^. Lastly, Col-III was reported to be co-expressed with Col-I to form heterotypic fibrils^38,39^, and to regulate the dimensions of the fibrils such as their diameter and periodicity^23,40,49,50^. Despite its interesting properties, Col-III has not previously been used for tissue engineering applications, to our knowledge. Thus, one original contribution of the present study was to investigate Col-III as the fibrillar component of the hydrogels^39,40,48,49^.

Although collagen scaffolds offer exceptional biological properties and thus have been widely developed and expanded for STE^18,22,32,51,52^, their scaffold compaction, high biodegradation rates and relatively poor mechanical properties have prompted the development of hybrid collagen-based scaffolds^52^ using other biomolecules, such as glycosaminoglycans^31,53^, elastin^54^ or chitosan^30,55–57^ as well as various cross-linking methods. Chitosan is a well-characterized linear positively charged polysaccharide derived from the partial deacetylation of chitin, the second most abundant natural biopolymer^58–61^. Intrinsic biocompatibility, similarities to glycosaminoglycans, minimal foreign body reaction, absence of chronic inflammatory response, low immunogenicity, immune enhancing effects, and finally abundance in nature and low production cost have made chitosan very appealing for tissue engineering, wound healing and drug delivery applications^11,62–66^. Unmodified chitosan is however poorly soluble, except in acidic solutions, due to its strong intermolecular hydrogen bonds^62^. Different chitosan derivatives have been investigated to overcome this drawback, among which glycol-chitosan (GCS), a 6-(2-hydroxyethyl) ether derivative of chitosan, was shown to be soluble over the entire pH range^67^.

The goal of the present study was to investigate the potential of a novel injectable hydrogel combining the advantages of Col-III, Col-I and GCS, with semi-interpenetrating polymeric networks (semi-IPN) of heterotypic collagen fibrils (Col-1&III) in GCS, as shown in Fig. 1c. This biomaterial is designated as Col-I&III/GCS (Col-I&III/G1 and Col-I&III/G2 for 2% and 1% final concentration of GCS, respectively). Col-III was introduced for previously mentioned reasons. Modulation of the Col-III content regulates the geometrical features of heterotypic fibrils with Col-I^23^. The proposed hydrogel can thus better be tuned to offer a tissue-mimetic microenvironment for both *in vivo* and *in vitro* STE applications. Unlike films, sponges or matrices, fibrillar collagen mimics the fibrillar structure of soft tissue ECM^52^. Only a few recent studies have focused on the incorporation of Col-I or collagen type II fibrils in composite hydrogels^68^. The present study is the first to propose hybrid hydrogels containing heterotypic Col-I&III fibrils. Here, collagen fibrils were formed during the fabrication procedure using a mixture of tropocollagen types I and III (Fig. 1b), and their presence was confirmed using atomic force microscopy (AFM) imaging.

Hydrogels of Col-III and GCS, designated as Col-III/GCS (Col-III/G1 and Col-III/G2), were also fabricated and characterized, as a specific case study of Col-I&III/GCS hydrogels with 0% Col-I (Table 1). The abundance of thick Col-I fibrils, the shortage of Col-III in scar tissue and the known role of Col-III in regulating scar formation suggest that Col-III/GCS hydrogels could be superior to Col-I based hydrogels for *in vivo* STE, especially for wound healing applications to help reduce scar formation. Scaffolds of Col-I and GCS, designated as Col-I/GCS (Col-I/G1 and Col-I/G2), and single GCS hydrogels, designated as CTL/GCS (CTL/G1 and CTL/G2), were used as positive and negative controls, respectively. Two GCS concentrations of 2% (G1s) and 1% (G2s) were tested (Table 1). The structural properties of the proposed hydrogels were characterized via atomic force microscopy, environmental scanning electron microscopy (ESEM), and micro-computed tomography (micro-CT). The swelling capacity, biodegradation and mechanical stability of the hydrogels were investigated. Cell-biomaterial interactions were examined using cell viability, metabolic activity, proliferation, cell adhesion, cell migration and cell morphology tests. The proposed hydrogels (Col-I&III/GCS) were shown to support cell adhesion and implantation, and they provided longer half-life than previously investigated HA-based hydrogels^2^. Col-III/GCS hydrogels were observed to support various aspects of cell implantation. The *in vitro* results generally support Col-III as a potential substitute for Col-I in composite collagen-based biomaterials.

**Table 1:** Study groups with final concentrations of glycol-chitosan (GCS), collagen types I and III (Col-I: collagen type I, Col-III: collagen type III, Col-I&III: the simultaneous presence of Col-I and Col-III and CTL: negative control.

Group ID	Glycol-chitosan	Collagen type I	Collagen type III
Col-I&III/G1	2.00%	0.04%	0.04%
Col-III/G1	2.00%	0.00%	0.04%
Col-I/G1 (Positive control)	2.00%	0.04%	0.00%
CTL/G1 (Negative control)	2.00%	0.00%	0.00%
Col-I&III/G2	1.00%	0.04%	0.04%
Col-III/G2	1.00%	0.00%	0.04%
Col-I/G2 (Positive control)	1.00%	0.04%	0.00%
CTL/G2 (Negative control)	1.00%	0.00%	0.00%

G1 and G2 represent final GCS concentrations of 2% and 1% (GCS to collagen ratios of 50 and 25), respectively.).

## Results

### Morphological characterization

**Figure 2:**
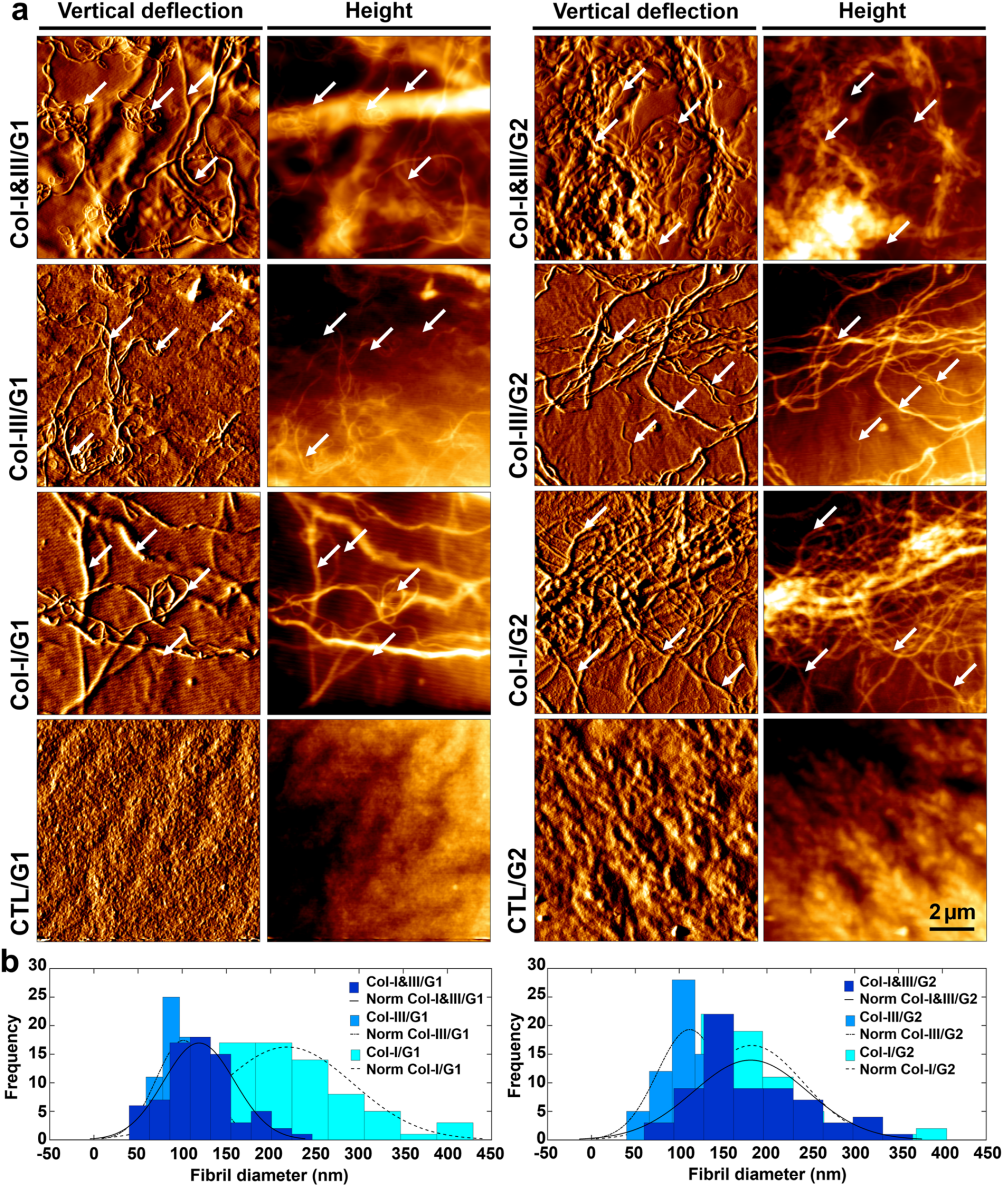
Atomic force microscopy (AFM) results. (**a**) Representative AFM images, vertical deflection and height images, of the proposed collagen (Col)/glycol-chitosan (GCS) hydrogels, Col-I&III/GCS and Col-III/GCS, positive controls (Col-I/GCS) and negative controls (CTL/GCS). Tropocollagen molecules self-assembled into fibrils during the fabrication process, and thus semi-IPN networks of fibrillar collagen structure within a GCS matrix were formed. Representative fibrils are identified using white arrows. No fibrillar structure was observed for the negative controls; (**b**) Histograms of the obtained fibril diameters. The mode was smaller for Col-III/GCS, compared with that of associated Col-I&III/GCS, which was itself smaller than that of associated Col-I/GCS (Col-III: collagen type III, Col-I: collagen type I, Col-I&III: the simultaneous presence of Col-I and Col-III, and CTL: negative controls. G1 and G2 represent final GCS concentrations of 2% and 1%, respectively).

Figure 2a shows representative AFM images of Col-I&III/GCS, Col-III/GCS, Col-I/GCS (positive control) and CTL/GCS (negative control) hydrogels. A semi-IPN network of fibrillar collagen structure is visible within the GCS matrix. Representative fibrils are indicated using white arrows. Figure 2b shows histograms of the measured fibril diameters. The mode (i.e., the peak of the histogram) was smaller for Col-III/GCS (≈100 and 110 nm for Col-III/G1 and Col-III/G2) than that of associated Col-I&III/GCS (≈125 and 175 nm for Col-I&III/G1 and Col-I&III/G2), which was itself smaller than that of associated Col-I/GCS hydrogels (positive control, ≈225 and 175 nm for Col-I/G1 and Col-I/G2). The mean ± standard deviation of the fibril diameters listed in Supplementary Table A.1 further confirmed these observations in terms of the average diameters.

**Figure 3:**
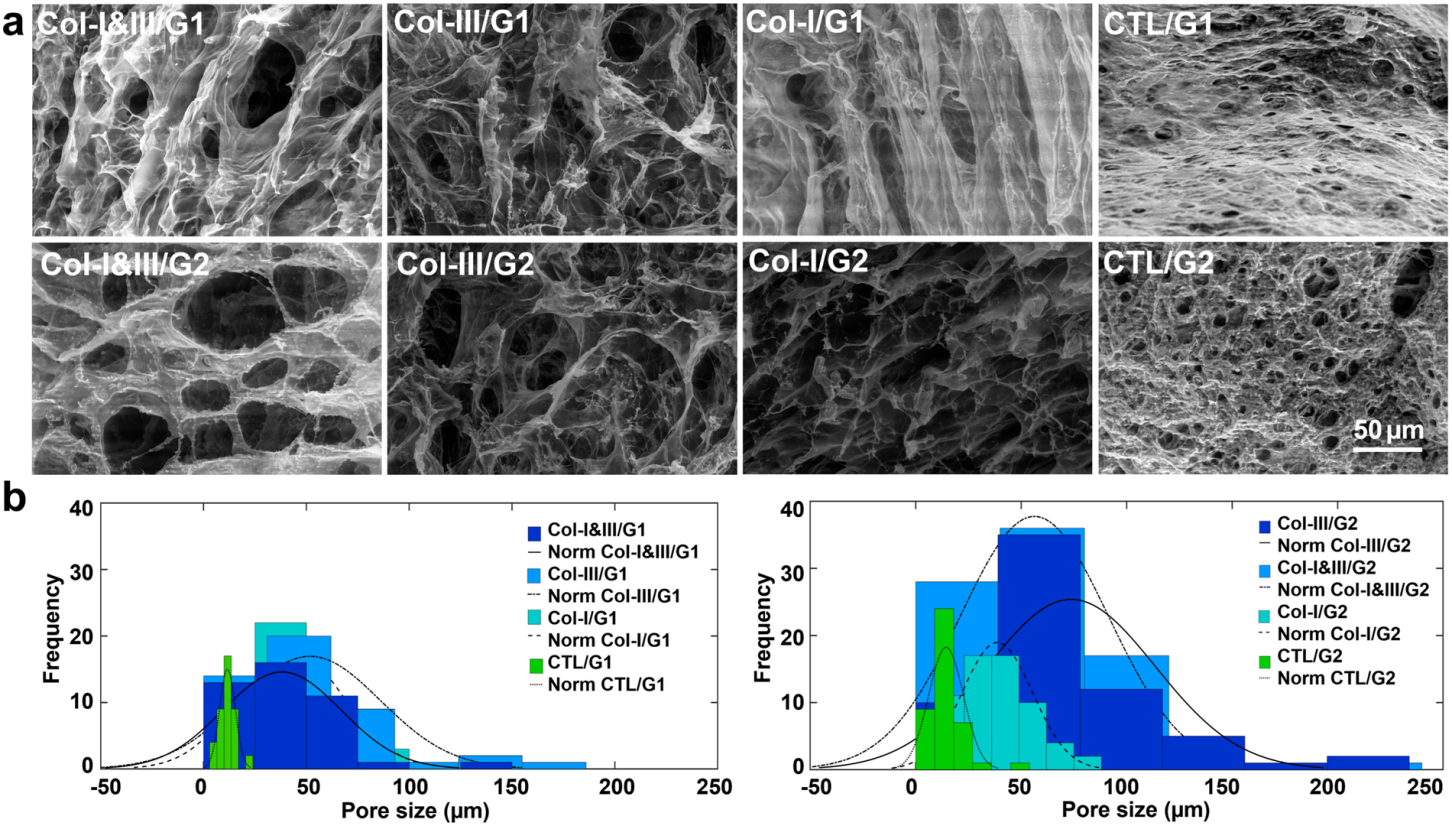
Environmental scanning electron microscopy (ESEM) results. (**a**) Representative ESEM images of Col-I&III/glycol-chitosan (GCS), Col-III/GCS, positive control (Col-I/GCS) and negative control (CTL/GCS) hydrogels. Porous structures with interconnected pores of irregular shapes were found for the Col/GCS hydrogels. Rounded closed pores with smaller dimensions were found for the negative controls. The pore size increased with the incorporation of collagen in GCS hydrogels; (**b**) Histograms of the measured pore sizes out of three replicates for each study group. The mode was slightly larger for Col-III/GCS hydrogels, compared with those of associated Col-I&III/GCS hydrogels and positive controls (Col-I/GCS), which were all greater than those of the associated negative controls (Col-III: collagen type III, Col-I: collagen type I, Col-I&III: the simultaneous presence of Col-I and Col-III, and CTL: negative controls. G1 and G2 represent the final GCS concentration of 2% and 1%, respectively).

Figure 3a shows representative ESEM images. Porous structures with interconnected pores of irregular shapes were observed in the hybrid hydrogels. In contrast, the negative controls exhibited rounded closed pores with significantly smaller dimensions. Figure 3b shows histograms of the pore sizes out of three replicates for each study group. The mode was slightly larger for Col-III/GCS (≈55 and 60 μm for Col-III/G1 and Col-III/G2) than that of associated Col-I&III/GCS (≈40 and 55 μm for Col-I&III/G1 and Col-I&III/G2) and Col-I/GCS (positive control, ≈37.5 and 40 μm for Col-I/G1 and Col-I/G2) hydrogels, which were all greater than that of associated negative controls (≈10 and 15 μm for CTL/G1 and CTL/G2). The mean ± standard deviation and the range of the measured pore size shown in the second and third columns of Supplementary Table A.2 further confirmed these observations. Micro-CT data showed a total porosity of greater than 90% for all the Col/GCS hydrogels.

### Swelling characterization and biochemical stability

**Figure 4:**
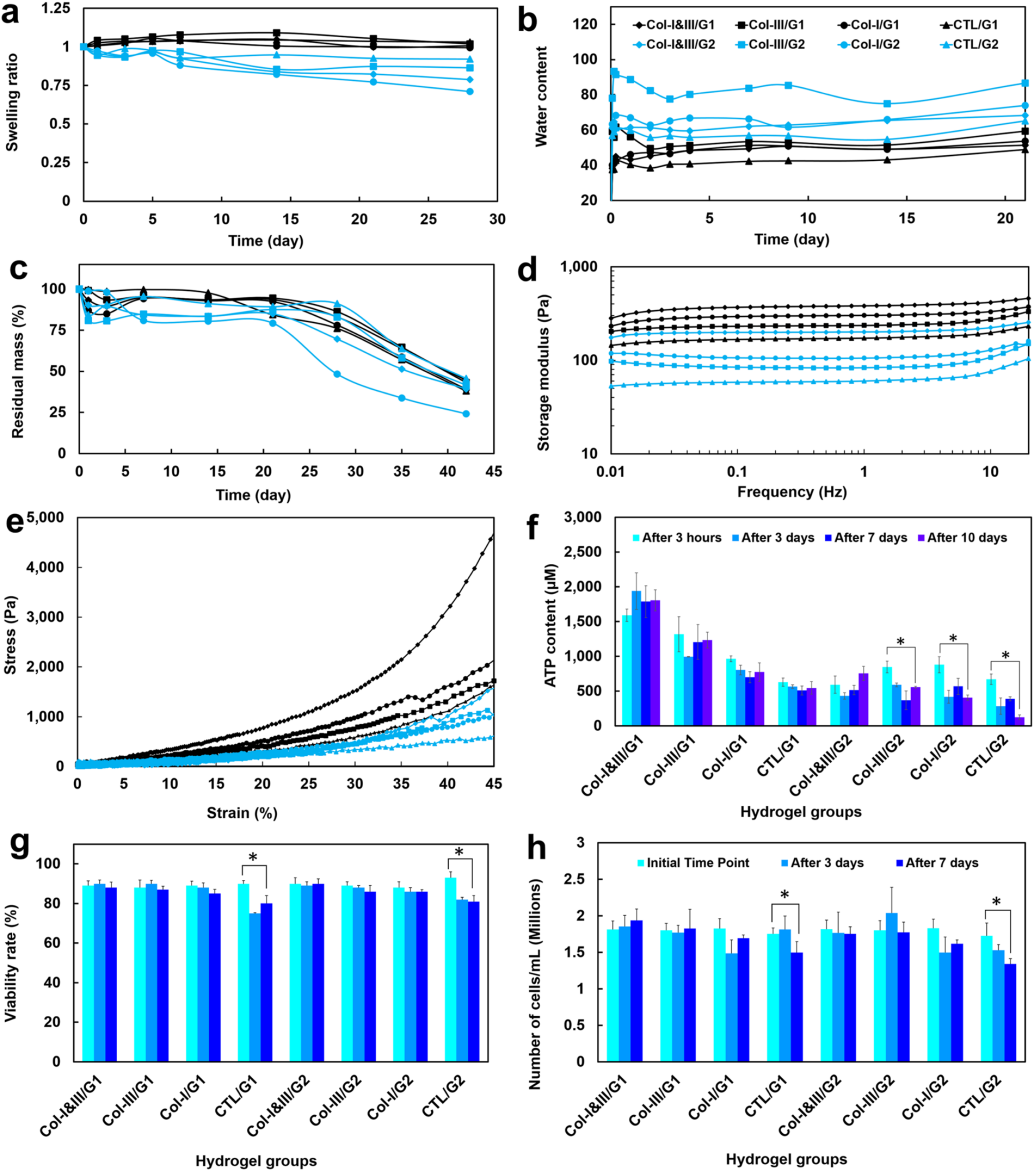
(**a**) Swelling kinetic of the collagen (Col)/glycol-chitosan (GCS) hydrogels initiated from the wet state. Greater swelling ratios were obtained for the Col/GCS hydrogels with higher GCS concentrations (G1s). *W*_*t*_ and *W*_0_ are the wet weights at the arbitrary and initial times, respectively; (**b**) Water content of the hydrogels initiated from the dried state. *W*_*t*_ and *W*_*d*_ are the weights at the swollen and dried states, respectively. A sudden increase was found in the water content of the hydrogels 2 hours after immersion in PBS. The hydrogels reached an equilibrium water content after 2 days; (**c**) The fractional residual mass (%) of the hydrogels immersed in the enzyme solution for a duration of 42 days. A half-life of more than 35 days was found for the proposed hydrogels; (**d**) Average storage moduli of the hydrogels for the frequencies between 0.01 and 20 Hz. Larger storage moduli were found for the Col/GCS hydrogels compared with the associated negative controls; (**e**) Stress-strain curves of the hydrogels obtained through compression test. The area inscribed below the graph denotes the compressive toughness of the sample; (**f**) The adenosine triphosphate (ATP) content of the cells over a duration of 10 days; and (**g,h**) Cell viability rates (%) and cell density (total number of cells per mL) in each hydrogel configuration over a duration of 7 days (Col-III: collagen type III, Col-I: collagen type I, Col-I&III: both Col-I and Col-III, and CTL: negative controls. G1 and G2 represent the final GCS concentration of 2% and 1%, respectively).

Figure 4a and b show the swelling kinetic of the proposed hydrogels in PBS initiated from the wet and dehydrated states, respectively. Greater swelling ratios were obtained for the hydrogels with higher GCS concentrations than the associated hydrogels with lower GCS content (G1s versus G2s), when initiated from the wet state, as shown in Fig. 4a. Upon immersion in PBS, the hydrogels with lower GCS concentration (G2s) started to degrade gradually, and the swelling ratio decreased slightly during the first day. The hydrogels reached their equilibrium state after one day.

Figure 4b shows the water content of the hydrogels over a duration of 21 days. A sharp increase was observed in the water content of the hydrogels 2 hours after immersion in PBS, which yielded wet weights of more than 40 and 50 times the associated initial weights for the hydrogels with 2% (G1s) and 1% (G2s) GCS, respectively. The hydrogels reached an equilibrium water content after 2 days. Greater water contents were obtained for the Col/GCS hydrogels than for the associated negative controls (CTL/GCS). Col-III/GCS hydrogels yielded greater water contents (≈59.1 and 78.1 for Col-III/G1 and Col-III/G2) than associated Col-I&III/GCS (≈38.2 and 61.2 for Col-I&III/G1 and Col-I&III/G2), the positive (≈40.1 and 63.3 for Col-I/G1 and Col-I/G2) and negative (≈37.5 and 56.9 for CTL/G1 and CTL/G2) controls. Figure 4c shows the residual mass percentages of the hydrogels immersed in the enzyme solution over a duration of 42 days. A half-life of more than 35 days was found for the hybrid hydrogels.

### Mechanical characterization

Figure 4d shows the average storage moduli of the hydrogels for the frequencies between 0.01 and 20 Hz. Larger storage moduli were obtained for the Col/GCS hydrogels compared with the associated negative controls. Also, greater storage moduli were found for the hydrogels with higher GCS level (G1s versus G2s). Col-I/GCS hydrogels were found to possess larger storage moduli than those of associated Col-III/GCS hydrogels. The storage and loss moduli for the frequencies of 1.0 and 10 Hz are listed in Table 2. The viscoelastic properties of the hydrogels slightly increased for the frequency of 10 Hz compared with the associated values for 1 Hz.

**Table 2:** Viscoelastic properties of the hydrogels.

Group ID	Storage modulus at 1 Hz (Pa)	Storage modulus at 10 Hz (Pa)	Loss modulus at 1 Hz (Pa)	Loss modulus at 10 Hz (Pa)
Col-I&III/G1	381.09 ± 15.77	415.72 ± 13.68	4.73 ± 0.28	13.67 ± 1.23
Col-III/G1	235.61 ± 11.60	261.85 ± 12.61	3.15 ± 1.01	9.35 ± 1.07
Col-I/G1 (Positive control)	301.48 ± 15.65	330.76 ± 18.48	2.25 ± 0.50	7.65 ± 1.44
CTL/G1 (Negative control)	172.46 ± 1.25	196.95 ± 3.21	2.01 ± 0.37	9.45 ± 2.42
Col-I&III/G2	200.84 ± 14.30	223.02 ± 10.25	1.86 ± 0.42	5.96 ± 1.33
Col-III/G2	83.15 ± 10.18	107.49 ± 8.90	1.61 ± 0.30	6.04 ± 0.49
Col-I/G2 (Positive control)	105.74 ± 10.87	128.95 ± 9.24	0.82 ± 0.10	4.21 ± 0.29
CTL/G2 (Negative control)	60.52 ± 5.14	84.36 ± 2.04	0.56 ± 0.08	3.36 ± 0.25

Storage and loss moduli were measured for the frequencies between 0.01 to 20 Hz. The representative data (mean± standard deviation) at 1 and 10 Hz is shown (Col-I: collagen type I, Col-III: collagen type III and Col-I&III: the simultaneous presence of Col-I and Col-III; G1 and G2 represent 2% and 1% final glycol-chitosan concentration, respectively.).

Figure 4e shows stress-strain curves from compression tests. Larger elastic moduli (not shown here) were obtained for the Col/GCS hydrogels than for the associated negative controls. Also, greater elastic moduli were found for the hydrogels with higher GCS level, as expected.

### Mechanical stability (fatigue)

The Col/GCS hydrogels were stable under continuous dynamic mechanical stimulation. The addition of Col-I and Col-III does not adversely influence the mechanical stability of the GCS matrix^65^.

### Cell viability and cell proliferation

Figure 4f shows the adenosine triphosphate (ATP) content of the cells encapsulated in the hydrogels after 3 hours, 3, 7 and 10 days. A slight decrease was observed in the ATP level after 3 days in the hydrogels for most of the study groups except Col-I&III/G1. The ATP content slightly increased after 10 days in culture compared to those obtained after 7 days for the Col-I&III/GCS and Col-III/GCS hydrogels. Figure 4g shows the viability rates at the initial time point, after 3 and 7 days in culture. The viability rates were greater than 85% for the hybrid hydrogels and the positive controls after 3 and 7 days, which were greater than those of the associated negative controls (i.e., about 80%) at these time points. The viability was greater than 88% for all samples at the initial time point. Figure 4h shows the total number of cells per mL (i.e., cell density) over one week in culture. The cell density decreased significantly in the negative controls.

### Two-dimensional cell adhesion and three-dimensional cell morphology

**Figure 5:**
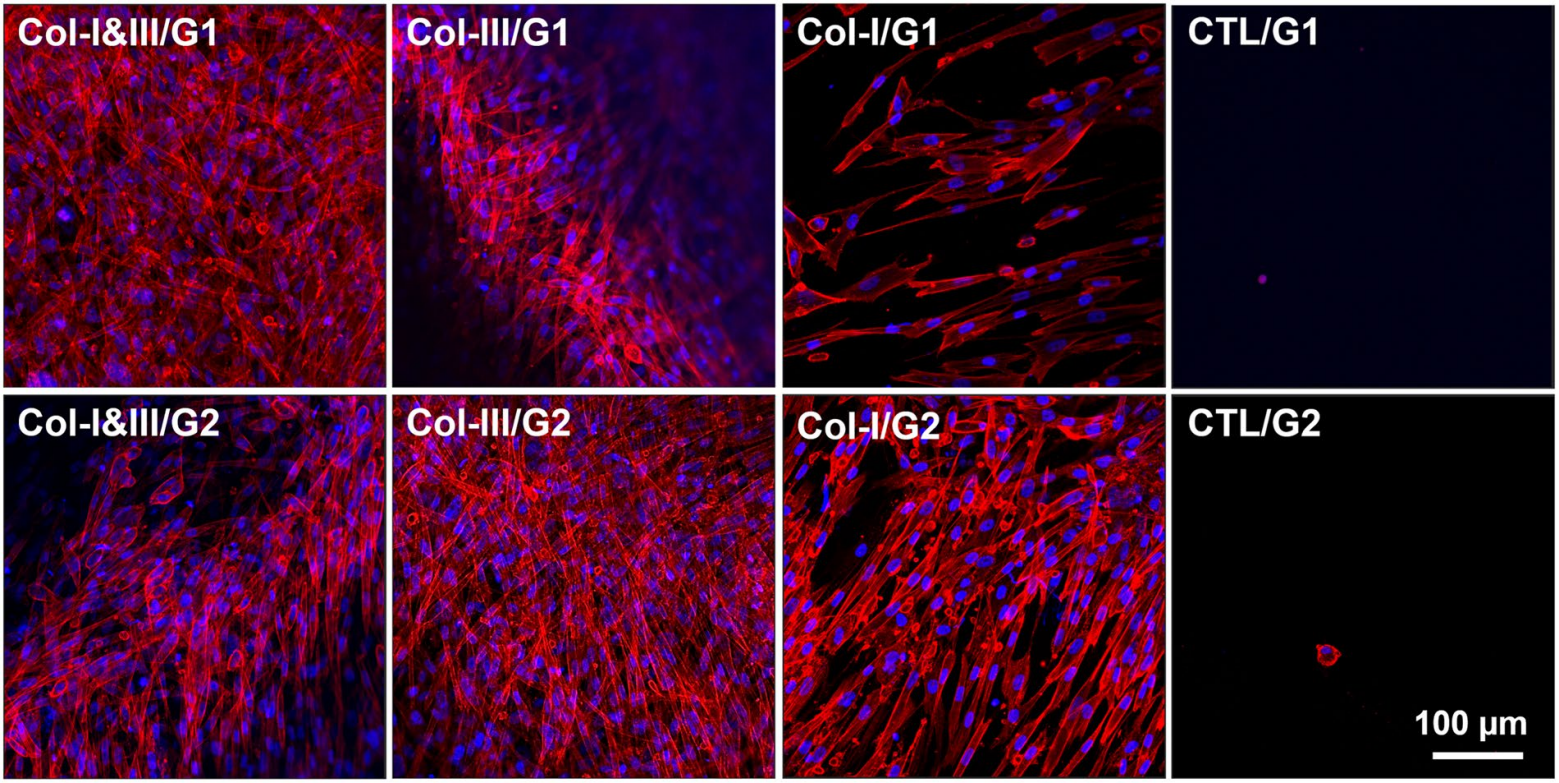
Representative fluorescent images of adherent cells cultured on the surface of the collagen (Col)/glycol-chitosan (GCS) hydrogels and negative controls. Actin cytoskeleton and nuclei are shown in red and blue, respectively. Fibroblasts were attached strongly to the surface of the proposed Col/GCS hydrogels. The cells were spread out extensively, and the formation of actin stress fibers were observed. On the contrary, only a few rounded cells were found attached on the surface of the negative controls after 3 days. The images were captured with a 20x objective, and the scale bar shows 100 μm (Col-III: collagen type III, Col-I: collagen type I, Col-I&III: both Col-I and Col-III, and CTL: negative controls. G1 and G2 represent the final GCS concentration of 2% and 1%, respectively).

Figure 5 shows representative fluorescent images of adherent cells cultured on the surface of the hybrid hydrogels and negative controls. Actin cytoskeleton and nuclei are shown in red and blue, respectively. Fibroblasts were attached strongly to the surface of Col-I&III/GCS and Col-III/GCS hydrogels as well as the positive controls. On the contrary, only a few rounded cells were found attached to the surface of the negative controls after 3 days.

**Figure 6:**
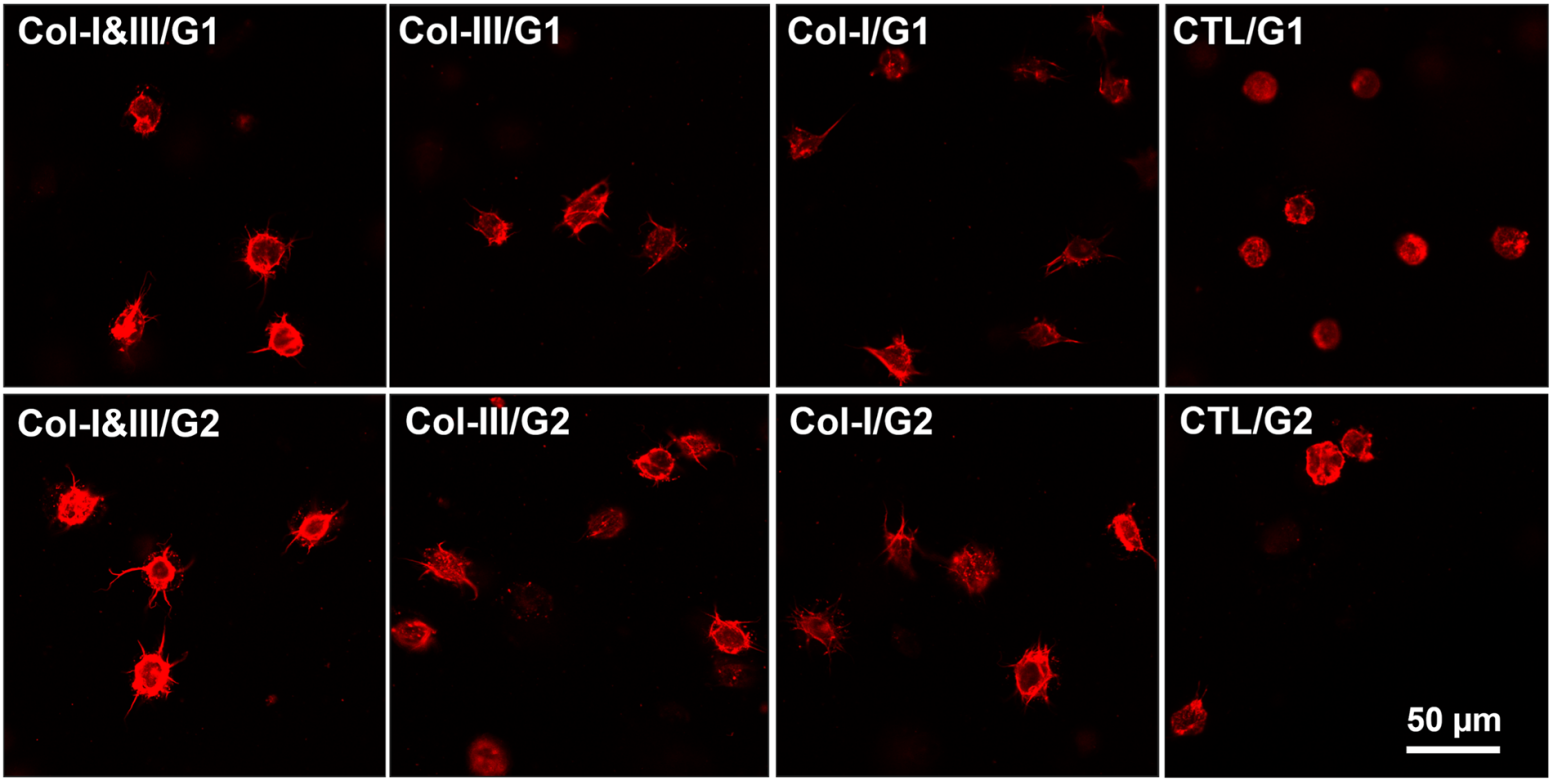
Representative fluorescent images of encapsulated cells in the collagen (Col)/glycol-chitosan (GCS) hydrogels and negative controls after 7 days in culture. The cells had a rounded shape at the initial time (not shown here). Growing actin stress fibers were found for cells cultured in the Col/GCS hydrogels, unlike those in the negative controls with a completely rounded shape. Larger actin fibers were found for the cells encapsulated in the hybrid hydrogels for 7 days compared with those cultured for only 2 days (Refer to Supplementary Fig. A.1). It was inferred that the cells adhered to the Col/GCS hydrogels.

Supplementary Fig. A.1 shows the actin cytoskeleton of the fibroblasts 2 days after encapsulation. Figure 6 shows the actin cytoskeleton after 7 days. The cells had a rounded shape at the initial time (not shown here). The cells encapsulated within Col-I&III/GCS and Col-III/GCS hydrogels started to form actin stress fibers at day 2, compared with those seeded in the negative controls with a completely rounded morphology. For the cells encapsulated in Col-I&III/GCS and Col-III/GCS hydrogels, actin stress fibers became longer after 7 days in culture, compared with the negative controls for which a rounded morphology was again observed. Our observations were consistent with the data shown in Table 3 in terms of chord diameters and cell ellipticity.

**Table 3:** Morphology data. Chord diameters (μm) and cell ellipticity were measured for cells encapsulated in the hydrogels after 2 and 7 days in culture.

Group ID	Chord diameter after 2 days (μm)	Chord diameter after 7 days (μm)	Ellipticity after 2 day	Ellipticity after 7 day
Col-I&III/G1	27.55 ± 4.01	35.50 ± 9.09	0.60 ± 0.24	0.62 ± 0.23
Col-III/G1	33.73 ± 7.45	40.56 ± 10.02	0.64 ± 0.20	0.69 ± 0.18
Col-I/G1 (Positive control)	25.46 ± 4.69	32.72 ± 4.75	0.68 ± 0.20	0.68 ± 0.18
CTL/G1 (Negative control)	15.82 ± 4.49	16.86 ± 1.54	0.83 ± 0.17	0.89 ± 0.10
Col-I&III/G2	28.61 ± 4.30	37.82 ± 4.43	0.65 ± 0.21	0.69 ± 0.15
Col-III/G2	30.68 ± 6.08	46.19 ± 9.78	0.62 ± 0.29	0.57 ± 0.23
Col-I/G2 (Positive control)	25.38 ± 1.87	33.25 ± 8.64	0.63 ± 0.23	0.56 ± 0.18
CTL/G2 (Negative control)	19.74 ± 1.84	20.80 ± 1.95	0.84 ± 0.15	0.81 ± 0.14

Mean ± standard deviation of the data is listed (Col-I: collagen type I, Col-III: collagen type III and Col-I&III: the simultaneous presence of Col-I and Col-III; G1 and G2 represent 2% and 1% final glycol-chitosan concentration, respectively.).

## Discussion

Col-III has been reported to be co-expressed with Col-I to form heterotypic fibrils in many soft tissues^38,39^ and was shown to play a crucial role in regulating fibril dimensions, such as their diameter^23,40,49,50^. Such changes in the geometry and composition of the collagen fibrils affect their mechanical properties and therefore the biological and mechanical functions of tissues. We have recently demonstrated that the incorporation of tropocollagen type III in tropocollagen type I yields the formation of heterotypic fibrils of both types I and III with smaller diameter, periodicity (Fig. 1a) and elastic moduli than those of single Col-I counterparts^23^. Col-III is also known to help regulate scar formation. It was previously reported that mature scar tissue has 50% less Col-III than normal tissue, and its fibers form thick bundles arranged in parallel arrays^24^. In the present study, we proposed the incorporation of both tropocollagen types I and III in the hybrid hydrogel. We hypothesized that the incorporation of these two fibrillar collagen types would result in a more tissue-mimetic structure than the previously studied collagen-based biomaterials containing only Col-I. A ratio of 1:1 for Col-III to Col-I tropocollagens was selected, which corresponds to that previously reported for liver and vocal fold tissues^23^. Injectable hydrogels specific to other soft tissues might be fabricated following the proposed procedure using the associated Col-III to Col-I ratios (See Fig. 1 in ref.^23^).

Chitosan mimics the glycosaminoglycan content of soft tissues such as vocal folds^55^. It can be physiologically depolymerized in the presence of human enzymes such as lysozyme, and therefore yields biodegradable scaffolds. GCS, soluble over the entire pH, was used to better support cell culture. GCS-based micro- or nano-carriers were shown to be effective for drug delivery, including for hydrophobic drugs. Unlike unmodified chitosan, GCS has been shown to yield super-porous hydrogel scaffolds with enhanced swelling capacity, due to the presence of highly hydrophilic glycol groups^67,69,70^. The introduction of chitosan in collagen scaffolds was shown to increase the overall matrix integrity by providing a more controlled biodegradation^57^. Porous collagen/chitosan hydrogels have been developed and characterized to combine the advantages and minimize the drawbacks of each constituent^71^. The addition of chitosan to collagen scaffolds was reported to influence the ultrastructure and yield larger pore size^57^. It was also reported that adherence and growth of cells were significantly enhanced in collagen/chitosan scaffolds^72^.

Chitosan hydrogels can be formed via covalent cross-linking of the chitosan derivatives using poly ethylene glycol diacrylate^73^, or aldehyde cross-linkers such as glutaraldehyde and glyoxal^65,74^, or by UV irradiation, and thermal variations^11^. Glutaraldehyde has been widely used to cross-link collagen- or chitosan-based scaffolds. However, it was shown that toxic monomeric glutaraldehyde was released upon the scaffold degradation, which was reported to yield adverse cellular effects both *in vitro* and *in vivo*^75^. Glyoxal is the smallest dialdehyde, which consequently results in releasing smaller quantities of aldehyde during degradation. It works as glycating agent in human tissues and body fluids in physiological conditions. This justifies the use of glyoxal as the cross-linking agent, in the present study. The concentration of glyoxal in the human blood is about 12.5 μg/ml (0.00125%), which is slightly smaller than what used here (0.005%). High concentrations of glyoxal (≥0.025%) causes cellular damage by oxidative stress. Recently, GCS hydrogels with different glyoxal concentrations (0.02, 0.015, 0.01, 0.0075, 0.005 and 0.0025%) were investigated for vocal fold engineering^65^, and no cytotoxic response was observed for glyoxal concentrations lower than or equal to 0.015%. The final concentration of 0.005% was found to be optimal^65^. The cross-linking reactions between glyoxal and GCS start as soon as they are mixed, which lowers the concentration of free glyoxal in the mixture and thus minimizes its cytotoxic effects. Furthermore, glyoxal is detoxified enzymatically through the cytosolic glyoxalase system^65^.

One target application for the present work is vocal fold engineering, discussed here as an example for STE. The most severe disorders affecting voice are those in which part of the soft lamina propria is lost or replaced by stiff fibrous tissue. Scarring is a common voice disorder caused by the surgical removal of benign or malignant vocal fold lesions, phono trauma or intubation over an extended period. Scarred vocal fold tissue is fibrotic with diminished elastin but excessive disorganized Col-I deposition^76^. In a rabbit model, it was shown that both stiffness and dynamic viscosity are around one order of magnitude larger in scarred vocal fold compared with the normal counterpart^77^. To ensure proper phonation, viscoelastic biomaterials are often injected to treat scarring via compensation of the stiffness of the scar tissue, or effective modulation of wound healing and regeneration of functional vocal fold tissue^78–80^. Detailed descriptions of previously investigated vocal fold-specific hydrogels are discussed elsewhere^81^. Briefly, HA derivatives and Col-I have been widely investigated for vocal fold tissue engineering^11,78,79^. These macromolecules are the main components of the vocal fold ECM. Native HA in its uncross-linked form provides a short half-life *in vivo*. The degradation rate of HA-based hydrogels by covalent cross-linking of the modified HA was found to yield lower degradation rates^79^. The introduction of gelatin, denatured collagen, into HA hydrogel was shown to improve adhesion and migration of fibroblasts while adversely increasing the hydrogel biodegradation^10,78^. Hahn *et al*. developed injectable collagen-based composite hydrogels with either HA or alginate, among which the collagen/alginate scaffolds resulted in lower degradation rates compared with the collagen/HA scaffolds^31^. Alginate-based scaffolds, however, were shown to offer poorly regulated degradation due to the absence of enzymes in mammals to break down alginate^82^. The use of polyethylene glycol tetra acrylate as the cross-linking agent for a composite injectable hydrogel composed of thiolated-HA and thiolated-Ge was investigated to overcome the fast degradation rate and to enhance the mechanical and biochemical stability of the scaffolds^2^. Although no cytotoxicity was observed *in vitro*, and the encapsulated cells synthetized Col-I and Col-III after 7 days in culture, our recent *in vivo* study in rabbits showed serious inflammatory response. Consequently, we recently turned our attention to the use of GCS for vocal fold engineering^65^. Although the *in vitro* characterizations were promising, the single GCS hydrogel does not support cell adhesion.

The AFM images (Fig. 2a) revealed the presence of collagen fibrils, and further supported our hypothesis that tropocollagen molecules self-assembled into fibrils during the fabrication process. A smaller average diameter was obtained for Col-I&III/GCS than that of the associated positive controls. These observations are consistent with the previously mentioned investigation on the *in vitro* fibrillogenesis of Col-I and Col-III^23^. The differences were found to be statistically significant (P < 0.05) between Col-I&III/GCS and Col-III/GCS hydrogels with the same GCS concentration, and between Col-I&III/G1 and Col-III/G1 hydrogels and the associated positive controls. Statistically significant differences were also observed in the fibril diameters between Col-I&III/G1 and Col-I&III/G2 hydrogels, i.e., with a decrease in the GCS concentration. A decrease in GCS concentration was then observed to result in an increase in the fibril diameter and the number of collagen fibrils. The characteristic periodicity of collagen fibrils was not apparent in the AFM images since the fibrils were embedded within the GCS matrix. The covalent cross-linking of the hydrogels provides strong covalent bonds between collagen fibrils and the GCS matrix. Thus, the semi-IPN fibrous structure was chemically stable over a wide temperature range. The hydrogels preserved their fibrillar structure at room temperature up to 24 hours after fabrication. It was observed that the fibrillar structure of the hydrogels is stable over a wide range of temperatures (4−37 °C). The *in vitro* fibrillation was performed through adjusting the pH from the primary acidic solution, pH of 2, to the physiological pH of 7.4. Collagen fibrils extracted from animal tissues are expensive and hard to access, and may trigger cross-species reactions^68^. We are not aware of Col-III fibrils extraction out of any animal tissues. Collagen fibril sources mostly provide Col-I or a mixture of collagen types I and III, for which the exact ratio of Col-III to Col-I is not determined. Here, we proposed the use of tropocollagen types I and III molecules, which are enzymatically extracted from the ECM secreted by the human neo-natal fibroblast cells. This source will decrease concerns related to cross-species complications.

Larger interconnected pores were obtained following the incorporation of collagen in the GCS matrix. The differences in the pore size were statistically significant (P < 0.05) between the negative controls (CTL/GCS) and associated Col/GCS hydrogels. A small increase in pore size was obtained due to a decrease in the concentration of GCS (G2s versus G1s) for Col-I&III/GCS, Col-III/GCS and CTL/GCS groups. But the differences were statistically insignificant (P > 0.05). The open pores found in Col-I&III/GCS and Col-III/GCS hydrogels provide more room for direct contact between nutrients, growth factors, gases and the encapsulated cells. The average pore sizes for the hybrid hydrogels were appropriate for fibroblast culture (≈45.6, 73.7, 51.8, 56.3, 42.2 and 38.7 μm for Col-I&III/G1, Col-I&III/G2, Col-III/G1, Col-III/G2, Col-I/G1 and Col-I/G2, respectively), compared with those of the negative controls (≈9.7 and 14.4 μm for CTL/G1 and CTL/G2). It should be noted that the microstructure of hydrogels might be significantly affected by the freeze-drying process, and thus these effects should be considered in interpreting the porosity data. All samples were dried simultaneously under identical circumstances to minimize the associated artifacts. Our findings were consistent with those of the quantitative Micro-CT analysis shown in the fourth column of Supplementary Table A.2. The total porosity of the hybrid hydrogels, in the wet state, was greater than 90%. This total porosity has been previously reported to provide appropriate diffusive transport within a cell-seeded scaffold *in vitro*^83^. Micro-CT 3D reconstruction revealed a homogenous distribution of pores in Col-I&IIII/GCS and Col-III/GCS hydrogels.

The swelling kinetics of the hydrogels improved following an increase in the chitosan to collagen ratio (50 and 25 for G1s and G2s)^56^. The water content of the hydrogels, the tendency of the dehydrated hydrogel to absorb the swelling agent, was investigated as another metric for their swelling kinetics. This parameter shows whether the pore size is larger in a wet state than a dehydrated state. The hybrid hydrogels yielded larger water contents than the associated negative controls, and the differences were statistically significant (P < 0.05). Interestingly, lower water contents were obtained for the hydrogels with higher GCS concentrations, when initiated from the dehydrated state. The Col-III/GCS hydrogels showed greater water contacts than those of the associated Col-I&III/GCS hydrogels, and the differences were statistically significant (P < 0.05). The Col-III/G2 hydrogel showed the best swelling response in terms of the water content property, which means it allows a larger increase in the pore size upon immersion in PBS, and thus better supports cell penetration and motion inside the scaffold. The nutrient and gasses transport would also be more effective for the hydrogels with higher water contents. Our data (78.1 for Col-III/G2) was consistent with previously published data reporting a water content of about 70 as the optimal value achieved for *in vitro* cell culture^84^. Phosphate buffer solution with pH of 7.4 was selected as the swelling agent to stimulate the same equilibrium condition as that of *in vivo* culture studies through providing the physiological pH level^30^.

The hybrid hydrogels had a half-life of more than 35 days and they featured a greater biochemical stability than that of the HA-based hydrogels previously used for STE with half-life of 2–3 weeks in cartilage, 3–5 days in vocal fold, less than one day in epidermis, and a few minutes in blood^2,85^. The hydrogels with higher GCS concentration (G1s) were more resistant to enzymatic degradation over the first 30 days than those with lower GCS concentration. The differences were statistically significant between Col-I&III/G1 and Col-I&III/G2 as well as between Col-III/G1 and Col-III/G2 hydrogels. No statistically significant differences were observed between the two negative control groups. Upon immersion in the enzyme solution, the hydrogels with lower GCS concentration (G2s) started to degrade gradually, and the residual mass was about 80% at day 14. A residual mass of more than 90% was obtained for the hydrogels with higher GCS content (G1s) at day 14. The negative controls in general showed higher biochemical stability over the first 14 days than the associated Col/GCS hydrogels. However, the differences were statistically insignificant between CTL/G1 and associated Col-I&III/G1 and Col-III/G1 hydrogels. The average residual mass for CTL/G1 was smaller than those of associated Col-I&III/G1 and Col-III/G1 hydrogels at days 21, 28 and 35. Col-I/G2 hydrogels showed the smallest residual mass (≈24%) at the final time point (42 days). The differences between the residual masses of Col-I/G2 hydrogel and those of Col-I&III/G2 and Col-III/G2 were statistically significant. The degradation rates of the hydrogels containing Col-I were greater than those of the associated counterparts containing Col-I&III, Col-III and the negative controls, which further shows that the incorporation of Col-III in the Col/GCS improves their biochemical stability.

The degradation rate of the scaffolding materials should mirror the regeneration rate of the host tissue, especially for *in vivo* applications^2,65,86^. Also, a controlled biodegradation is appropriate for *in vitro* studies using tissue culture bioreactors, for which the hydrogel provides the scaffold for the cells to reside, interact with, and thus synthesize, deposit and remodel neo-tissue^2^. A hydrogel with a controlled biodegradation characteristic will preserve its structural integrity over a determined duration, and thus provide the encapsulated cells with an appropriate microenvironment to sense and respond to the biomechanical and biochemical stimuli^4^. The longer residence of Col-III/GCS and Col-I&III/GCS hydrogels will provide constant interactions between the scaffold and the patient’s cells, such as macrophages and fibroblasts, which may help modulate wound healing towards scar-free tissue especially considering providing Col-III in the wound site. Also, Col-I&III/GCS and Col-III/GCS hydrogels can be used as scaffolds for long-term *in vitro* investigations. In terms of vocal fold engineering, to date, no standard criterion exists to evaluate hydrogel biodegradation. A total degradation time between one and three months is however required. This time correlates with the end of the inflammation period, which varies between individuals^65^. We assumed that a half-life of more than one month, which provides longer *in situ* residence compared with that of the currently used HA-based hydrogels, thereby decreases the need for regular reinjections currently performed for voice recovery^2^. High molecular weight HA is known to improve cell migration, and facilitate wound healing processes^85^; however, the fast enzymatic biodegradation of HA requires intensive cross-linking that yields heavily cross-linked networks, which may not support cell migration. Chitosan was previously reported to be more resistant to degradation and to offer better biochemical stability^65^, which was further confirmed through a comparison between the hybrid hydrogels and an optimized HA-based hydrogel previously investigated using similar *in vitro* conditions^2^.

A rapid gelation time is a desired feature of injectable hydrogels to support their integrity *in situ* and to prevent them from flowing into the neighbouring tissue. The addition of collagen to the GCS matrix led to a significant decrease in their rheometer-determined onset of gelation time (i.e., the transition time when the solution phase changes from liquid-state to solid-state). The time sweep data (not shown here) showed a rapid onset of gelation time of a few seconds for Col-I&III/GCS and Col-III/GCS hydrogels, which was significantly smaller than that of about two minutes for the negative controls. It was also found impossible to extract Col-I&III/G1 and Col-I/GCS hydrogels out of the vial after one minute, which further showed that these hydrogels are mostly cured at this point. This time was about three, ten, three, five and over twenty minutes for Col-III/G1, CTL/G1, Col-I&III/G2 and Col-I/G2, Col-III/G2, and CTL/G2 hydrogels. The storage moduli of Col-I&III/GCS hydrogels were within the range of those of the very soft tissues such as human vocal fold lamina propria^87^. The viscoelastic properties of the tissue are known to regulate cellular physiology and the associated tissue hemostasis. As expected, the Col-I/GCS hydrogels were found slightly stiffer than the associated Col-III/GCS hydrogels. Col-I fibrils are known to be stiffer than Col-III fibrils^23^. Mechanical properties of various tissues have been shown to correlate with the viscoelastic properties of the associated structural collagen fibrils. Larger storage moduli were found for Col-I&III/GCS hydrogels than for associated Col-I/GCS and Col-III/GCS hydrogels. This group of hydrogels has a higher total concentration of collagen, as shown in Table 1.

The area inscribed below the stress-strain curve obtained through compression test denotes the compressive toughness of the hydrogels^88^. As Fig. 4e illustrates, Col/GCS hydrogels were found to have larger compressive toughness compared with the negative controls. Also, Col-I&III/G1 was found to have a larger compressive toughness than Col-I/G1. Similarly, Col-I/G1 showed a larger compressive toughness than Col-III/G1. Further, hydrogels with lower GCS concentration showed lower values of compressive toughness. Hydrogel scaffolds may fail under dynamic cyclic loading, which thus limits their applications for engineering mechanically active soft tissues such as heart valves and vocal folds. It was recently reported that HA hydrogels cross-linked with poly ethylene glycol diacrylate were fragmented following continuous dynamic loading in a vocal fold bioreactor^2^. Also, it was shown elsewhere that GCS hydrogels were mechanically stable under continuous cyclic loading in the same bioreactor^65^. Here, it was found that the addition of Col-I or Col-III caused no adverse effects on the mechanical stability of the hydrogels.

No cytotoxicity response was observed for the HVFFs encapsulated within the proposed hydrogels. The viability rates were greater than 85% for all the Col/GCS hydrogels. However, the viability rates decreased in the negative controls after 7 days, and the differences were statistically significant (P < 0.05) between the initial time point and after 7 days in culture. The viability data was in agreement with the ATP results. Greater cell metabolic activity was found for the cells encapsulated in the Col/GCS hydrogels compared with those in the negative controls at each time point. It was also observed that the ATP content decreased significantly for the negative controls, especially for the CTL/G2 group. The incorporation of Col-I&III, Col-III or Col-I resulted in a slight increase in the viability rates compared with the negative controls, which was consistent with the known biological effects of collagen in supporting cell culture. Furthermore, no statistically significant differences were observed in terms of cell viability between Col-I&III/GCS, Col-III/GCS and Col-I/GCS hydrogels. It was previously shown that GCS hydrogels with high concentrations of glyoxal (≥0.01%) exhibited cytotoxicity to human fibroblasts, and thus resulted in lower cell viability. The glyoxal concentration used in the present study, 0.005%, however, did not cause any cytotoxic effects^65^.

As shown in Fig. 4h, the total number of cells per mL remained relatively constant for Col-I&III/GCS and Col-III/GCS hydrogels from the initial time to day 7, with statistically insignificant differences over time or between hydrogels with different GCS concentrations. The cell density decreased for the positive controls, Col-I/GCS hydrogels, from the initial time to day 3. A slight increase was observed after 3 days in culture, with statistically insignificant differences between the initial time, day 3 and day 7. The cell density decreased for the negative controls, and the differences were statistically significant between the initial time point and day 7. Our data is consistent with previous findings on the cell proliferation of vocal fold fibroblasts in collagen-HA and collagen-alginate hydrogels, which showed relatively constant cell numbers over a duration of 28 days^31^. It was previously reported that the incorporation of chitosan in collagen hydrogels inhibited cell proliferation of K526 cells compared to those cultured in pure collagen gels, and the cell numbers decreased in the collagen-chitosan gels^57^. In the present study, we did not observe any significant decrease in the cell density for Col-I&III/GCS and Col-III/GCS hydrogels.

A large number of HVFFs were found attached to the surface of the hybrid hydrogels (Fig. 5), and the differences between the population of adherent cells on the surface of the hybrid hydrogels and that of the negative controls were significant (greater than a hundredfold). Unlike natural ECM components such as collagen, most cells do not have receptors to natural or synthetic polymers^4^. For instance, GCS does not provide any cell-binding sites, and thus does not support cell attachments to the surface of the negative controls. Col-I is recognized for providing binding sites for cell attachments, especially fibroblasts^4,9,51^. Here, we showed that the addition of Col-III or a combination of collagen types I and III (Col-I&III) to GCS hydrogels improves their cell adhesion property significantly. This is important for the development, healing and engineering of mechanically active tissues such as heart valves and vocal folds. Cells that are poorly attached to the substrate cannot sense mechanical stimuli, such as tension and vibrations^89^. HVFFs had a spindle-like shape on the surface of the hybrid hydrogels, as shown in Fig. 5. They were spread over the surface of Col/GCS hydrogels, and the formation of actin stress fibers were observed. In contrast, only one or two rounded cells were found for the negative controls. Interestingly, after 3 days in culture on the surface of the Col/GCS hydrogels, HVFFs were found to form a layer of more than 60 μm thick (from the top of the cell layer towards the bottom of the well, Supplementary Fig. A.2). It might be inferred that the cells started to move into the hydrogels.

Growing actin stress fibers were found for the cells encapsulated in the Col/GCS hydrogels after 2 days in culture. In contrast, those in the negative controls had a completely rounded morphology (Supplementary Fig. A.1). This confirms that the cells started to attach and grow within the hybrid hydrogels at this time^30^. Actin stress fibers were found to grow longer for the cells in Col-I&III/GCS and Col-III/GCS hydrogels after 7 days in culture. Again a rounded morphology was obtained for the cells in the negative controls. Although the morphology was not completely spindle-shape after 7 days, the formation and growth of actin stress fibers showed that the cells adhered to the hybrid hydrogels, and their morphology was changing towards a fibroblast-like morphology over time. As shown in Table 3, larger chord diameters and smaller cell ellipticity data were observed for the cells encapsulated in the Col/GCS hydrogels after 2 and 7 days in culture, compared with those in the negative controls, and the differences were statistically significant (P < 0.05) at both these time points. Smaller cell ellipticity for the cells in the Col/GCS hydrogels further proved that their morphology was changing from a rounded morphology at the initial time toward a more fibroblast-like morphology. The significantly larger ellipticity obtained for the cells in the negative controls showed that their morphology was close to a rounded one in every imaged plane. As part of our future work, we will study the changes in the morphology over 28 days^31^.

The average and standard deviation of overall displacement and speed of the cells encapsulated in the hydrogels over a duration of 12 hours are shown in Supplementary Fig. A.3. Larger displacements were found for the cells in the hydrogels with larger GCS concentration (G1s vs. G2s). Histograms of the overall displacement and speed (not shown here) for the cells encapsulated in different groups of the hydrogels showed that a larger percentage of cells moved a distance greater equal to 20 μm in the Col/GCS hydrogels compared with those within the negative controls. This might be due to the presence of the collagen network, to which cells adhere and anchor themselves. A larger percentage of cells in the Col/GCS hydrogels moved at a maximum speed greater or equal to 0.15 μm per minute than those in the negative controls. These results further confirmed that cells could move inside the hybrid hydrogels. Cell recruitment, infiltration and migration will be investigated in more details, as part of our future work.

As our future direction, gene and protein analysis will be performed to investigate and customize the application of the hybrid hydrogels for engineering particular soft tissues. Also, the cell-seeded Col-I&III/GCS and Col-III/GCS hydrogels will be cultured in a phono-mimetic bioreactor^2^, and the long-term effects of phonation-induced mechanical stimulation on the cells’ behavior in terms of cell proliferation, morphology and ECM production will be investigated. *In vitro* studies will be designed to study the behavior of macrophages in the proposed hydrogel. Finally, a comprehensive animal study will be conducted using a rabbit model to examine the long-term outcome of the Col-I&III/GCS and Col-III/GCS hydrogels *in vivo*.

## Concluding Remarks

A novel tissue-mimetic hybrid injectable hydrogel of collagen fibrils in a glycol-chitosan matrix was developed and characterized. Tropocollagen types I and III molecules were fibrillated during the fabrication procedure. Heterotypic collagen fibrils were incorporated with tissue-specific Col-III to Col-I ratios. Col-III was introduced for the first time as a key structural component of the hydrogels. The presence of collagen fibrils was confirmed using AFM. Large interconnected open pores were found in the hybrid hydrogels compared with the negative controls with small rounded close pores.

Larger water contents were obtained for Col-I&III/GCS hydrogels than those of the negative controls. The swelling kinetics of the hydrogels improved following an increase in the GCS to collagen ratio. A half-life of more than 35 days was obtained for Col-I&III/GCS and Col-III/GCS hydrogels immersed in the enzyme solution that shows an encouraging biochemical stability compared with that of the HA-based hydrogels previously used for vocal fold engineering. It was also observed that an increase in the GCS concentration resulted in an increase in the overall matrix integrity. The storage moduli of the hybrid hydrogels were within the range of those of very soft tissues, such as human vocal folds. Greater compressive toughness was found for Col-I&III/GCS hydrogels than the negative controls. It was also found that Col-I&III/GCS hydrogels were mechanically stable under continuous cyclic loading. The hybrid hydrogels thus appear to be good candidates for the engineering of mechanically challenged soft tissues which undergo complex dynamic biomechanical stimuli. The hybrid hydrogels were found to support cell implantation in terms of cell viability, metabolic activity, adhesion and migration. The formation of actin stress fibers was observed for the fibroblasts encapsulated within Col-I&III/GCS and Col-III/GCS hydrogels after 2 days in culture. Cells encapsulated within the negative controls had a rounded morphology after 7 days in culture.

Col-III/GCS hydrogels, a specific case of Col-I&III/GCS hydrogels, also seemed suitable for *in vivo* STE, which might help prevent scar formation or at least modulate scarring via the known influence of Col-III in modulating scar formation, its effects on the heterotypic fibril morphological properties and through enhancing Col-III levels at the wound site. The Col-III/GCS hydrogels were shown to support cell culture and cell adhesion. Col-I&III/GCS and Col-III/GCS provide biomimetic microenvironments for cell implantation, and are potential candidates for STE, specially to engineer mechanically challenged soft tissues both *in vivo* and *in vitro*.

## Methods

### Cell culture in flask

Human vocal fold fibroblasts (HVFFs) were cultured in a mixture of Dulbecco’s Modified Eagle Medium (Life Technologies Inc., Burlington, ON), 10% fetal bovine serum (Sigma-Aldrich Corporate, St. Louis, MO), 1% penicillin/streptomycin (Sigma-Aldrich Co.), 1% sodium pyruvate (Life Technologies Inc.), and 1% MEM non-essential amino acids (Sigma-Aldrich Co.) at 37 °C, in 5% CO_2_ humidified atmosphere. All concentrations for the cell culture medium (CCM) ingredients are expressed as %v/v (volume-to-volume percentage). The CCM was replaced every three days. Cells were disassociated using 0.25% trypsin-EDTA when they reached around 70% confluency. Cells were subsequently centrifuged and suspended with serum-free CCM.

### Preparation of the collagen/glycol-chitosan hybrid hydrogels

GCS and glyoxal were purchased from Sigma-Aldrich Corporate. Human tropocollagen type I (3 mg/mL in 0.01 N HCl, pH 2) and type III (1 mg/mL in 0.01 N HCl, pH 2) were provided by Advanced BioMatrix Inc., Carlsbad, CA. Solutions of GCS 5% and glyoxal 10% in 1x phosphate-buffered saline (PBS; PBS refers to 1x PBS through the whole manuscript.) were prepared, and placed in a laboratory mixing rotator (Glas-Col Corporate, Terre Haute, IN) at 30 revolutions per minute (RPM) for 24 consecutive hours. The solutions were subsequently autoclaved to prevent contamination.

Eight different groups of cell-seeded hydrogels were prepared to obtain target concentrations of 2 × 10^6^ cells/mL and 0.005% glyoxal and different concentrations of GCS, Col-I and Col-III, as listed in Table 1. All concentrations for the hydrogel ingredients are expressed as %w/v (weight-to-volume percentage). Figure 1b shows a schematic of the step-by-step fabrication procedure. The required amounts of 5% GCS and tropocollagen solutions were added to an eppendorf vial, and the mixture was vortexed for one minute. The pH of the solution was then adjusted using HCL 0.1 N to ensure a physiological pH balance (i.e., 7.4 ± 0.1) in the final cell-seeded hydrogel. HVFFs, dispersed in serum-free CCM, were added to the mixture after 2 hours. Subsequently, the cross-linking agent (glyoxal) was added, and the solution was gently pipetted up and down a few times to homogenize the mixture. The positive and negative controls were prepared following the same procedure.

### Atomic force microscopy (AFM)

A JPK atomic force microscope (AFM, JPK Nano-wizard@3 Bio-Science, Berlin, Germany) was used to image the hydrogel samples, as made and 24 hours after fabrication, to identify the formation of collagen fibrils during the fabrication process. A 10 μL drop of the hydrogel mixture was placed on a microscope glass slide immediately after fabrication, and cured in the form of a thin film. After 24 hours, 200 μL of PBS was added on the hydrogel film for 30 minutes to hydrate it. A silicon nitride cantilever (MLCT Micro-cantilever, Bruker, Mannheim, Germany) with defined spherical tip of radius 2 nm, and a Nanotools CONTR B50 cantilever (Nanotools USA LLC, Henderson, NV) with defined spherical tip of 50 nm ±10% were used to image three samples of each group. The National Institute of Health ImageJ software was used to measure the diameters of the collagen fibrils.

### Environmental scanning electron microscopy (ESEM)

A FEI Quanta 450 environmental scanning electron microscope was used to observe the morphology of the hydrogels^90^. The samples were prepared in the form of 1 mL cylindrical specimens in 2 mL eppendorf vials, which were then placed inside a 37 °C incubator for 24 hours. The samples were then frozen in liquid nitrogen for 3 minutes. They were subsequently freeze-dried for 48 hours, at −105 °C and 16 mTorr using a BenchTop K freeze-dryer (VirTis, SP Industries, Warminster, PA). The specimens were then cross-sectioned, and their surface morphology was observed at 10 kV and 10 mA.

### Micro-computed tomography (Micro-CT)

Micro-CT was performed using a SkyScan 1072 (SkyScan, Kontich, Belgium). The samples were prepared and incubated at 37 °C for 24 hours. They were then analyzed through a 360° flat-field corrected scan at 49 kV and 205 μA, with a rotational step size of 0.45°, a cross-sectional pixel size of 11.25 μm and no filter. The volumetric reconstruction (NRecon software, SkyScan) was performed with a beam hardening correction of 12, a ring artifact correction of 20, and an “auto“ misalignment correction. The 2D and 3D analyses were carried out using CTAn software and a grayscale intensity range of 50–85 (8 bit images) to remove background noise.

### Swelling characterization and *in vitro* enzymatic biodegradation

The swelling ratios were determined by immersing the samples in PBS (pH 7.4)^56^ at 37 °C with gentle mechanical stimulation (75 RPM). At pre-determined time intervals, the excess PBS on the hydrogel surface was removed using filter paper, and the wet weight of the swollen hydrogel was measured. The swelling ratio of the hydrogels was determined as *W*_*t*_/*W*_0_, in which *W*_*t*_ and *W*_0_ are the measured wet weights at the arbitrary and initial time, respectively.

To measure the water content of the hydrogels, pre-weighed freeze-dried samples were immersed in PBS and incubated at 37 °C with gentle mechanical stimulation (75 RPM). At pre-determined time intervals, the excess PBS was removed, and the wet weight of the samples were measured. The water content of the hydrogels was calculated as (W_*t*_ − W_*d*_)/W_*d*_, in which W_*t*_ and W_*d*_ are the measured weights at the swollen and dried states, respectively.

To investigate the *in vitro* biodegradation of the hydrogels, pre-weighed samples were immersed in the enzyme solution of PBS containing 13 μg/mL lysozyme (Sigma-Aldrich Co.) and 20 U/mL collagenase (Worthington Biochemical Corporation, Lakewood, NJ)^91–94^. The samples were incubated at 37 °C with gentle mechanical stimulation over a period of 42 days. The enzyme solution was replaced every two days. At pre-determined time intervals, the enzyme solution was removed, and the samples were washed three times for 10 minutes each (3 × 10 minutes) with PBS. The samples were then freeze-dried and weighed. The fractional residual mass of the hydrogels was obtained using W_*dt*_/W_*d*0_ × 100, in which W_*dt*_ and W_*d*0_ are the dry weights at each time point and at the initial time, respectively.

### Oscillatory shear rheometry

A TA Instrument Rheometer, Discovery Hybrid HR-2 (New Castle, DE), with a standard steel parallel-plate geometry of 20 mm diameter was used. The samples of volume 350 μL were loaded onto the bottom plate as a liquid and then the superior plate was lowered to obtain the desired gap of 1000 μm while still in the liquid phase^95^. A time sweep test was performed at a frequency of 1 Hz, an oscillatory strain of 10% and at 37 °C for a duration of one hour immediately after hydrogel fabrication to monitor the *in-situ* gelation of the hydrogels. Oscillatory frequency sweep tests were performed at 37 °C and an oscillatory strain of 10% over the frequency range between 0.01 and 20 Hz to determine the storage and loss moduli at different frequencies.

### Compression test

The rheometer was further used to perform mechanical compression test. Cylindrical hydrogel samples were fabricated and kept inside a tissue culture incubator for one hour to ensure they were fully cured and also to minimize alterations in the mechanical behaviour due to dehydration. The samples were then inserted between the plates of the rheometer. Compression test was conducted at a strain rate of 10 μm per second until the strain of 50%.

### Mechanical stability (fatigue) test

The hydrogels, listed in Table 1, were injected into the cavities of a phono-mimetic vocal fold bioreactor^2^, which mimics the complex dynamic biomechanical stimulation including the repeated loading-unloading cycles and collision forces between the two vocal folds during phonation. The bioreactor was then phonated continuously for 72 hours. Subsequently, the hydrogels were harvested and their mechanical integrity was evaluated visually following previous practice^2^. The fatigue test was repeated three times for each hydrogel composition.

### Cell viability and cell proliferation

Cell-seeded hydrogels were fabricated in petri dishes and incubated at 37 °C. CCM was replaced every 3 day. Samples were collected and snap-frozen 3 hours, 3, 7 and 10 days after fabrication. To investigate the metabolic activity of the cells encapsulated within the hydrogels, an ATP assay kit (Molecular Probes Inc., Oregon, USA) was used following the manufacturer’s instructions. The luminescence was determined in opaque-walled 96-well plates in a SpectraMax M5 microplate reader (Molecular Devices, Sunnyvale, CA). The ATP content was quantified using a standard curve.

Cell-seeded hydrogels were fabricated in 8-well tissue culture plates and incubated at 37 °C. CCM was replaced every day. At the initial time point, 3 days and 7 days after fabrication, the samples were stained using a LIVE/DEAD^®^ viability/cytotoxicity kit (Life Technologies Inc., Burlington, ON) following previous practice^2^. An inverted confocal fluorescence microscope (LSM710, Zeiss, Jena, Germany) was used to image the stained samples. All the images were acquired using a 20x objective (20x/0.8 Dry Plan-Apochromat, Zeiss). A series of xy images, forming a z-stack, were collected using a voxel size of 0.145 μm, 0.145 μm, and 10.000 μm along the x-, y- and z- directions, respectively. The resolution of the acquired images was 1024 × 1024 pixels coded in 12 bits for each color channel. Image acquisition and image analysis, including 3D reconstruction, were performed using the software Zen (Zeiss) and Imaris version 7.5.6 (Bitplane, South Windsor, CT, USA), respectively, as described in details elsewhere^2^. The viability rate was then calculated as the number of live cells divided by the total number of cells (i.e., the number of both live and dead cells) in each 3D image.

For cell proliferation analysis, cell-seeded hydrogels were harvested at the initial time point, 3 days and 7 days after fabrication. The samples were frozen in liquid nitrogen and kept at −80 °C. Cell proliferation was assessed using a Quant-iT ^TM^ PicoGreen ^®^ dsDNA assay kit (Thermo Fisher Scientific, Waltham, MA). The hydrogels were suspended in the extraction buffer (1 N NH_4_OH and 0.2% triton X-100 in deionized water), and treated with a SCILOGEX D160 homogenizer (SCILOGEX, LLC., Rocky Hill, CT) while kept on ice. The homogenized samples were then incubated with the PicoGreen working solution in 1x TE buffer (10 mM Tris-HCl and 1 mM EDTA, pH 7.5, 1:200) for 5 minutes in darkness. An amount of 200 μL of the obtained mixture was loaded in 96-well plates, and the fluorescence of the samples was measured using a SpectraMax M5 microplate reader (Molecular Devices, Sunnyvale, CA) at the excitation and emission wavelengths of 488 and 525 nm, respectively.

### Cell adhesion and cell morphology

For cell adhesion analysis, hydrogel films were fabricated at the bottom of 8-well tissue culture plates and incubated at 37 °C. After 24 hours, HVFFs with a concentration of 1 × 10^6^ cells/mL were cultured on the surface. After 3 days, actin cytoskeleton staining was performed using an actin cytoskeleton and focal adhesion staining kit (FAK100, EMD Millipore Headquarters, Billerica, MA). Cells were first fixed using 4% paraformaldehyde in PBS for 20 minutes. They were then permeabilized with 0.1% Triton X-100 in PBS for 5 minutes at room temperature. After washing the samples for 2 × 5 minutes using a buffer (PBS containing 0.05% Tween-20), they were blocked using 1% bovine serum albumin (BSA) in PBS for 30 minutes. The samples were then incubated with TRITC-conjugated phalloidin (1:250 in 1% BSA in PBS) for one hour. They were then washed 3 × 10 minutes. Nuclei were counterstained by DAPI incubation for 5 minutes, followed by washing for 3 × 10 minutes. The samples were subsequently imaged using the LSM710 confocal fluorescence microscope, as discussed for the cell viability test.

For cell morphology analysis, the 3D cell-seeded hydrogels were incubated at 37 °C. They were stained using the same staining procedure as for the adhesion test, at two days and seven days after preparation. The stained samples were imaged using the LSM710, following the procedure as for imaging the cell viability samples. Briefly, the images were acquired using a 20x objective. For each sample, z-stacks were collected using a voxel size of 0.145 μm, 0.145 μm, and 25.000 μm along the x-, y- and z- directions, respectively. Image analysis, including 3D reconstruction, were performed in Imaris (Bitplane Inc.). Particularly, the channels were separated, and the largest chord diameter of each cell and its ellipticity were measured using the red channel associated to the actin filaments. The images were thus closely evaluated both visually and semi-quantitatively^30^.

### Cell migration

For cell migration investigations, cell membrane was stained using Vybrant^®^ DiD cell-labeling solution (Life Technologies Inc.) prior to encapsulating the cells in the hydrogels, as described elsewhere^65^. Cell-seeded hydrogels were then fabricated in 8-well tissue culture plates and incubated at 37 °C. The samples were then imaged using the LSM710 microscope for 12 consecutive hours. All the images were acquired using a 10x objective. Image analysis, including 3D reconstruction, was performed using Imaris (Bitplane).

### Statistical analysis

Statistical significance was determined using a paired Student’s t-test, when applicable. Differences were considered significant at P < 0.05.

### Data availability statement

The datasets generated and analyzed during the current investigation are available from the corresponding author on reasonable request.

## Electronic supplementary material

Supplementary information
