# Supplementary material for: A tissue-mimetic nano-fibrillar hybrid injectable hydrogel for potential soft tissue engineering applications
Source: Sci Rep. 2018 Jan 18;8:1047. doi: 10.1038/s41598-017-18523-3 (PMC5773686; doi:10.1038/s41598-017-18523-3)
Supplement: Supplementary file 1 — Supplementary information [file 41598_2017_18523_MOESM1_ESM.pdf]

Supplementary materials for:
A tissue-mimetic nano-fibrillar hybrid injectable hydrogel for potential soft tissue engineering applications

Neda Latifi,[1] Meisam Asgari,[1] Hojatollah Vali,[2] and Luc Mongeau[1]

[1]*Department of Mechanical Engineering, McGill University, 817 Sherbrooke street west, Montreal, QC H3A 0C3, Canada*

[2]*Department of Anatomy & Cell Biology, McGill University, 3640 University street, Montreal, QC H3A 2B2, Canada*

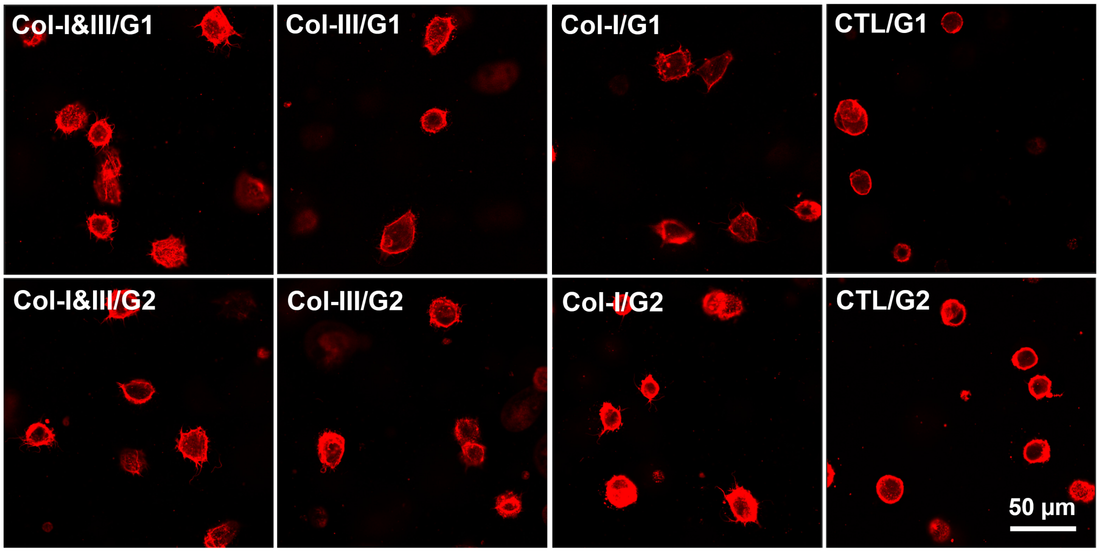

FIG. A.1: Representative fluorescent images of encapsulated cells in the collagen (Col)/glycol-chitosan (GCS) hydrogels and negative controls after 2 days in culture. The cells had a rounded shape at the initial time (not shown here). The cells encapsulated within the Col/GCS hydrogels started to form actin stress fibers at day 2, compared with those in the negative controls with a completely rounded morphology. It was inferred that the cells started to attach and grow in the Col/GCS hydrogels.

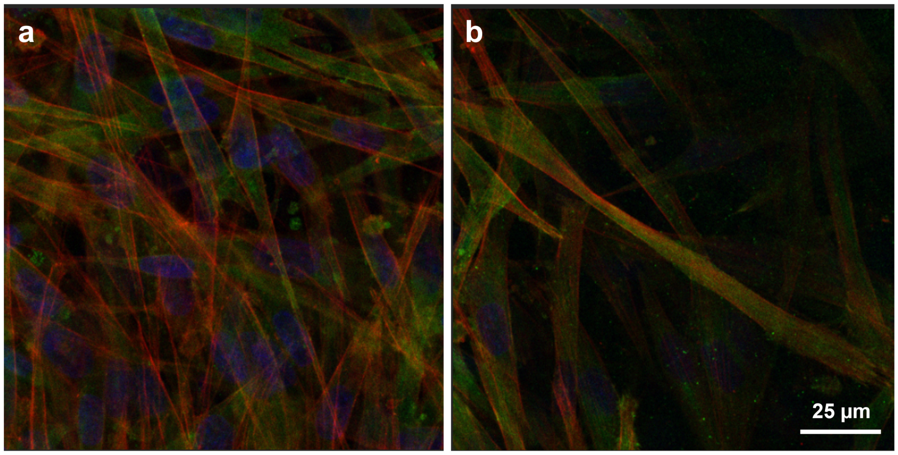

FIG. A.2: Representative fluorescent images of adherent cells cultured on the surface of Col-I&III/GCS hydrogels. (a) The top layer of the cells; (b) The bottom layer of the cells towards the bottom of the well. The two slides were about 60 μm apart in the z- direction. Focal contacts in cells, actin cytoskeleton and nuclei are shown in green, red and blue, respectively. The images were captured with a 63x oil objective, and the scale bar shows 25 μm (Col-III: collagen type III, Col-I: collagen type I, and Col-I&III: both Col-I and Col-III.

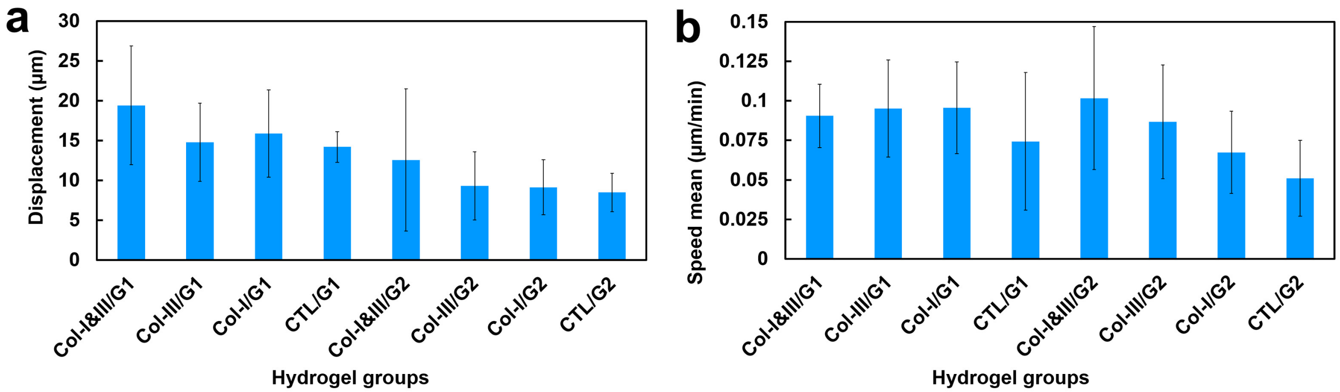

FIG. A.3: Cell migration results. (a) The overall displacement; and (b) the mean speed of the cells encapsulated in the hybrid hydrogels and the negative controls over a duration of 12 hours.

Group ID	Fibril diameter (nm)	Diameter range (nm)
Col-I&III/G1	119.1 ± 40.0	51–243
Col-III/G1	101.2 ± 30.7	46–210
Col-I/G1 (Positive control)	220.7 ± 66.9	110–424
Col-I&III/G2	181.4 ± 65.1	74–361
Col-III/G2	111.2 ± 37.1	54–274
Col-I/G2 (Positive control)	182.6 ± 57.1	96–399

TABLE A.1: Collagen fibril diameters (nm) obtained from the atomic force microscopy (AFM) images. Fibril diameters (mean ± standard deviation) and the diameter ranges are listed (Col-I: collagen type I, Col-III: collagen type III and Col-I&III: the simultaneous presence of Col-I and Col-III; G1 and G2 represent 2% and 1% final glycol-chitosan concentration, respectively.).

Group ID	Pore size (μm)	Pore size range (μm)	Total porosity (%)
Col-I&III/G1	45.6 ± 25.5	8.0–144.8	95.6 ± 0.7
Col-III/G1	51.8 ± 34.3	13.0–185.3	94.1 ± 0.8
Col-I/G1 (Positive control)	42.4 ± 25.4	9.8–148.1	95.3 ± 0.7
CTL/G1 (Negative control)	9.7 ± 5.9	3.4–40.8	87.2 ± 1.5
Col-I&III/G2	73.7 ± 39.8	18.8–229.7	95.8 ± 0.6
Col-III/G2	56.3 ± 35.1	10.0–235.9	98.4 ± 0.4
Col-I/G2 (Positive control)	38.7 ± 16.7	10.3–86.4	94.5 ± 0.7
CTL/G2 (Negative control)	14.4 ± 8.3	4.1–51.3	90.0 ± 0.9

TABLE A.2: Pore size of the hydrogels (μm) obtained from the environmental scanning electron microscopy images. Pore size (mean ± standard deviation) and the size ranges are listed (Col-I: collagen type I, Col-III: collagen type III and Col-I&III: the simultaneous presence of Col-I and Col-III; G1 and G2 represent 2% and 1% final glycol-chitosan concentration, respectively.). Micro-computed tomography data showed a total porosity of greater than 90% for all the collagen/glycol-chitosan hydrogels, which was previously reported to provide appropriate diffusive transport within a cell-seeded scaffold *in vitro*.
